# Indole-3-Acetic Acid Alleviates Nonalcoholic Fatty Liver Disease in Mice via Attenuation of Hepatic Lipogenesis, and Oxidative and Inflammatory Stress

**DOI:** 10.3390/nu11092062

**Published:** 2019-09-03

**Authors:** Yun Ji, Yuan Gao, Hong Chen, Yue Yin, Weizhen Zhang

**Affiliations:** Department of Physiology and Pathophysiology, Peking University Health Science Center, Beijing 100191, China

**Keywords:** indole-3-acetic acid, NAFLD, steatosis, oxidative stress, inflammation, lipid metabolism

## Abstract

Recent evidences have linked indole-3-acetic acid (IAA), a gut microbiota-derived metabolite from dietary tryptophan, with the resistance to liver diseases. However, data supporting IAA-mediated protection against nonalcoholic fatty liver disease (NAFLD) from an in vivo study is lacking. In this study, we assessed the role of IAA in attenuating high-fat diet (HFD)-induced NAFLD in male C57BL/6 mice. Administration of IAA (50 mg/kg body weight) by intraperitoneal injection was found to alleviate HFD-induced elevation in fasting blood glucose and homeostasis model assessment of insulin resistance (HOMA-IR) index as well as plasma total cholesterol, low-density lipoprotein cholesterol (LDL-C), and glutamic-pyruvic transaminase (GPT) activity. Histological examination further presented the protective effect of IAA on liver damage induced by HFD feeding. HFD-induced an increase in liver total triglycerides and cholesterol, together with the upregulation of genes related to lipogenesis including sterol regulatory element binding-protein 1 (Srebf1), steraroyl coenzyme decarboxylase 1 (Scd1), peroxisome proliferator-activated receptor gamma (PPARγ), acetyl-CoA carboxylase 1 (Acaca), and glycerol-3-phosphate acyltransferase, mitochondrial (Gpam), which were mitigated by IAA treatment. The results of reactive oxygen species (ROS) and malonaldehyde (MDA) level along with superoxide dismutase (SOD) activity and glutathione (GSH) content in liver tissue evidenced the protection of IAA against HFD-induced oxidative stress. Additionally, IAA attenuated the inflammatory response of liver in mice exposed to HFD as shown by the reduction in the F4/80-positive macrophage infiltration and the expression of monocyte chemoattractant protein-1 (MCP-1) and tumor necrosis factor-α (TNF-α). In conclusion, our findings uncover that IAA alleviates HFD-induced hepatotoxicity in mice, which proves to be associated with the amelioration in insulin resistance, lipid metabolism, and oxidative and inflammatory stress.

## 1. Introduction

Nonalcoholic fatty liver disease (NAFLD), the most common chronic liver disease worldwide, has been known to be inherently associated with obesity, insulin resistance, and dyslipidemia [1]. NAFLD is defined as the presence of hepatic steatosis on liver biopsy that is not initiated by alcohol consumption or other reasons (e.g., drugs, toxins, infections) [2]. Currently, the prevalence rate of NAFLD is estimated to be 24% around the world, among which 5–20% of patients with simple steatosis progress to nonalcoholic steatohepatitis (NASH) [3,4]. NASH is characterized by steatosis with lobular inflammation and the ballooning of hepatocytes, and increased risk of fibrosis, cirrhosis, and hepatocellular carcinoma [5].

Although the precise mechanisms related to NAFLD pathology are not completely elucidated, a multiple-hit (lipid accumulation, oxidative stress, endoplasmic reticulum stress, insulin resistance, gut microbiota, inflammatory response, dietary and genetic factors) hypothesis has been proposed to explicate the factors connecting the pathological process [6,7]. Insulin resistance and overloading of lipids in hepatocytes result in steatosis, oxidative damage, and inflammation in liver tissue [8,9]. The excessive accumulation of triglycerides aggravates the generation of reactive oxygen species (ROS), which disrupts the redox homeostasis by inducing oxidative stress and activates inflammatory signaling-mediated pro-inflammatory responses [10,11]. Hence, exploring endogenous and exogenous molecules that contribute to enhancing the systemic antioxidative and anti-inflammatory capacity will be beneficial to the recovery of NAFLD.

A large amount of evidence has revealed that the gut microbiota plays vital roles in regulation of NAFLD via producing bacterial metabolites such as short-chain fatty acids, indole and its derivatives, secondary bile acids, and trimethylamine [12,13,14,15,16]. Dietary tryptophan can be metabolized into indole-3-acetic acid (IAA) by gut microbiota through indole-3-acetamide pathway under the catalysis of tryptophan monooxygenase and indole-3-acetamide hydrolase [17]. A previous in vitro study demonstrated that IAA possessed the ability of scavenging free radicals [18,19]. Additionally, results from cultured cell lines of macrophages and hepatocytes indicated that IAA mitigates pro-inflammatory cytokine production from macrophages exposed to endotoxin and attenuates lipogenesis in hepatocytes induced by cytokine and free fatty acids [20]. However, no available data regarding the protective effects of IAA against NAFLD has been reported in an in vivo study. Thus, in the present study, the effects of IAA on high-fat diet-induced NAFLD, in particular, lipid metabolism disorder, oxidative and inflammatory stress in hepatic tissue were evaluated in a mouse model.

## 2. Materials and Methods

### 2.1. Chemicals

Indole-3-acetic acid was purchased from Sigma (St. Louis, MO, USA). Goat serum was bought from ZOMANBIO Biotechnology Co. (Beijing, China). An ELISA kit for mouse insulin was purchased from ExCell Biotech Co. (Taicang, China). Plasma total triglyceride and cholesterol kits were purchased from Biosino Bio-Technology and Science Inc. (Beijing, China). Antibodies against F4/80 (EMR1) were obtained from ABclonal (Woburn, MA, USA). Fluorescein (FITC)-conjugated secondary antibodies were obtained from Huaxingbio Biotechnology Co. (Beijing, China). Hematoxylin and eosin were procured from Zhongshan Jinqiao Biotechnology Co. (Beijing, China). Low-density lipoprotein cholesterol (LDL-C) and high-density lipoprotein cholesterol (HDL-C) kits were procured from Nanjing Jiancheng Bioengineering Institute (Nanjing, China). Kits used for the detection of glutamic oxaloacetic transaminase (GOT), glutamic pyruvic transaminase (GPT), total antioxidant capacity (T-AOC), malonyldialdehyde (MDA), catalase (CAT), superoxide dismutase (SOD), glutathione (GSH), and oxidized glutathione (GSSG) were bought from Solarbio Science & Technology Co. (Beijing, China). Trizol reagent, and kits for BCA protein assay, ROS detection, tissue total triglyceride and cholesterol assay were procured from Applygen Technologies Inc. (Beijing, China). GoScript™ Reverse Transcription System was from Promega (Madison, WI, USA). Hieff ^TM^ qPCR SYBR Green Master Mix was purchased from Yeasen Biotechnology Co. (Shanghai, China).

### 2.2. Animals and Treatments

4-week-old male C57BL/6 mice were obtained from Weitonglihua Experimental Animal Tech Co. (Beijing, China). All the animals were raised at a temperature of 22 ± 2 °C under a 12-h light/12-h dark cycle. During the experimental period, all the mice had free access to pelleted feeds and autoclaved drinking water. After two weeks of acclimatization, a total of 36 mice were randomly divided into four groups: normal chow diet (NCD, 10% kcal from fat) + vehicle group; normal chow diet (NCD) + IAA group; high-fat diet (HFD, 60% kcal from fat (D12492; Research Diets, New Brunswick, NJ, USA)) + vehicle group; and high-fat diet (HFD) + IAA group. Mice received IAA at a dose of 50 mg/kg body weight or vehicle daily for nine weeks by intraperitoneal injection after a three week exposure to NCD or HFD feeding. The dose of IAA used in the present study was according to the previous report [21] and our preliminary study and it was safe for the mice. IAA stock solution (10 mg/mL) was prepared in phosphate buffered saline (PBS) and was entirely dissolved by adding NaOH (1 N), followed by a pH adjustment to 7.4 with 25% (*v*/*v*) HCl. Body weight and food intake was recorded daily. The mice were fasted overnight and sacrificed after 12 weeks of NCD or HFD feeding and blood and liver samples were obtained. The concentrations of plasma IAA were detected by an ELISA kit (Cloud-Clone Corp., Houston, TX, USA) in mice from NCD + vehicle, NCD + IAA, HFD + vehicle, and HFD + IAA group. These were 118.2 ± 44.7, 125.1 ± 34.4, 110.8 ± 29.4, and 129.9 ± 38.7 ng/mL (mean ± standard deviation; *n* = 8–9), respectively. No significant difference was found between the groups, suggesting the elevation in level of blood IAA was transient following the administration of IAA. All the experimental processes were approved by the Animal Care Committee of Peking University Health Science Center and conducted strictly in conformity with the Guide for the Care and Use of Laboratory Animals of the Chinese Association for Laboratory Animal Science and Use. Efforts were made to minimize the number of animals and the suffering involved in this research.

### 2.3. Biochemical Analysis

Plasma were obtained from whole blood samples by centrifugation at 1600 g for 15 min at 4 °C. Small amounts of blood were collected from the tail tip and used to determine the fasting blood glucose by glucometers. Plasma insulin was measured by using an ELISA kit (ExCell Biotech) following the manufacturer’s instructions. The homeostasis model of assessment for insulin resistance (HOMA-IR) index calculated as the following equations: HOMA-IR = [fasting insulin (mU/L)] × [fasting plasma glucose (mmol/L)]/22.5 [22]. Total triglycerides and cholesterol levels in plasma were determined by the GPO/PAP method. The intensity of the color from the final product quinone imine at 490 nm was proportional to the concentration of total triglycerides and cholesterol in plasma. The supernatant of the tissue homogenate was used to determine total triglycerides and cholesterol in hepatic tissue by enzymatic methods according to the commercial kits. Plasma HDL-C and LDL-C were measured by a two-step reaction method. In brief, the lipoproteins that were not needed to be determined were eliminated from the first reaction. Then, the remaining HDL-C or LDL-C produced color by an enzymatic reaction in the second step, which could be quantified by measuring the absorbance at 546 nm. Plasma activity of glutamic oxaloacetic transaminase (GOT) and glutamic pyruvic transaminase (GPT) were evaluated by catalytic reaction that generated pyruvic acid. Pyruvic acid reacted with 2,4-dinitrophenylhydrazine (DNPH) to form 2,4, dinitrophenylhydrazone, which presents as a brownish red colour in an alkali condition. The enzymatic activity could be calculated by the optical density value obtained from the measurement of absorbance at 505 nm.

### 2.4. Histopathological Examination

Hepatic tissue was excised and washed with ice-cold phosphate-buffered saline (PBS), and then fixed in 4% paraformaldehyde for 24 h. The tissues were dehydrated using solutions of increasing alcohol concentration ranging from 70% to 100%, followed by transparentizing and paraffin wax processing. The tissue sections with 5 μm thickness were prepared for the following deparaffinage and hematoxylin and eosin (H&E) staining procedure. Samples were observed and visualized by an Olympus CKX53 inverted microscope (Olympus, Tokyo, Japan).

### 2.5. Quantitative Real-time Polymerase Chain Reaction (RT-qPCR)

The total RNA from hepatic tissues were extracted by using TRIzol regent. The RNA samples (4 µg) were reverse transcribed to cDNA using a GoScript™ Reverse Transcription System following the instructions provided by the manufacturer. Quantitative PCR (qPCR) was performed on an AriaMx Real-Time PCR system (Agilent Technologies, CA, USA) in a final volume of 20 µL containing upstream and downstream primers, Hieff qPCR SYBR Green Mater Mix, and cDNA template. The amplification procedure was as follows: 95 °C for 5 min, 40 cycles of 95 °C for 10 s, 60 °C for 20 s, and 72 °C for 24 s. The relative mRNA expression for targeted genes were normalized by reference gene GAPDH and were calculated by 2^−ΔΔCT^ method. The sequences of primers used for RT-qPCR are listed in Supplementary Table S1.

### 2.6. Analysis of Oxidative Stress Indexes

The ROS level was determined by 2′,7′-dichlorofluorescein diacetate (DCFH-DA) assay. Briefly, homogenate of liver tissue in PBS was subjected to 10 μM DCFH-DA probe. After a 30 min incubation at 37 °C in dark, fluorescence intensity was measured by a SpectraMax Gemini-EM microplate reader with excitation and emission wavelengths of 488 and 525 nm, respectively. Total protein concentration in tissue homogenate was determined by using the BCA method. The absorbance of the mixture was recorded at 562 nm. The detection of T-AOC in plasma was based on the reduction of Fe^3+^-TPTZ to Fe^2+^-TPTZ by antioxidants under an acid condition. The total antioxidant capacity of samples can be calculated by the detection of absorbance of the blue color from Fe^2+^-TPTZ. The MDA level was measured through the generation of red MDA-TBA (thiobarbituric acid) adduct. Catalase activity in the tissue homogenates were determined by the decomposition reaction with hydrogen peroxide (H_2_O_2_), which results in a reduction in the absorbance at 240 nm. SOD activity was quantified by the inhibition of formazan formation from nitroblue tetrazolium, which was converted by superoxide anion yielded from a xanthine-xanthine oxidase reaction system. The quantification of GSH relied on the formation of a yellow colored product (5-thio-2-nitrobenzoic acid) generated from the reaction of the sulfhydryl group of GSH with DTNB (5,5′-dithio-bis-2-nitrobenzoic acid). On this basis, GSSG could be quantified by using glutathione reductase and 2-vinylpyridine.

### 2.7. Immunofluorescence Histochemistry

To visualize the glycoprotein F4/80 on the cell surface, liver histologic sections were blocked with 1% normal goat serum in PBS for 1 h at room temperature and then incubated with the primary antibody for F4/80 (1:100 in 1% normal goat serum) at 4 °C overnight in a humidified box. After washing with PBS three times, the sections were stained with FITC-conjugated goat anti-rabbit secondary antibody (1:50 in 1% normal goat serum) for 1 h at room temperature in a dark place, followed by washing with PBS and Hoechst 33342 staining (10 μg/mL) for 5 min. The images were captured by using a microscope coupled with a U-RFL-T fluorescence microscopy unit (Olympus, Tokyo, Japan).

### 2.8. Statistical Analysis

All the results are expressed as mean ± standard error of the mean. Statistical analysis was conducted using the GraphPad Prism 7.0 software (San Diego, CA, USA). Multiple comparison analysis was performed by one-way ANOVA followed by the Student–Newman–Keuls test. A *p*-value < 0.05 was considered as statistical significance.

## 3. Results

### 3.1. Indole-3-Acetic Acid (IAA) Ameliorates High-Fat Diet-Induced Systemic Insulin Resistance

**Figure 1:**
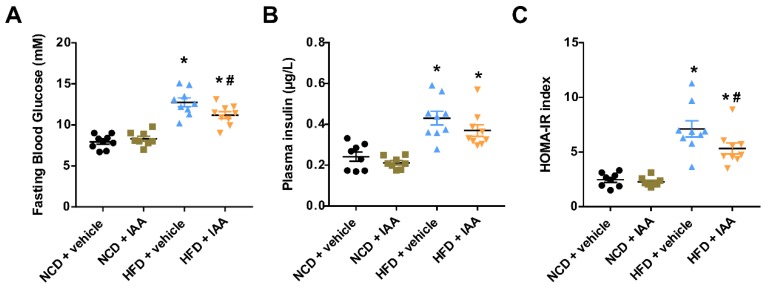
Indole-3-acetic acid (IAA) protected against high-fat-diet (HFD)-induced insulin resistance in mice. (**A**) Fasting blood glucose levels determined by a glucometer in mice fasted overnight. (**B**) Results from ELISA for plasma insulin. (**C**) HOMA-IR calculated as described in the Materials and Methods section. Data are expressed as the mean ± standard error of the mean. *n* = 8–9. * *p* < 0.05 vs. NCD + vehicle; ^#^ *p* < 0.05 vs. HFD + vehicle.

Average daily feed consumption was not affected by the IAA injection (Supplementary Figure S1A). Meanwhile, daily energy intake was equivalent among the four groups (Supplementary Figure S1B). After 12 weeks of feeding, mice subjected to the high-fat diet (HFD) showed body and liver weight significantly higher than normal chow diet (NCD)-fed mice (*p* < 0.05). However, IAA treatment for 9 weeks did not alter body or liver weight compared with vehicle-treated mice fed with HFD (Supplementary Figure S1C,D). The fasting blood glucose of mice that received the HFD diet was significantly higher than that of the NCD group (*p* < 0.05), while this effect was alleviated by IAA treatment (*p* < 0.05) (Figure 1A). HFD feeding induced an increase in the level of plasma insulin (*p* < 0.05) (Figure 1B). IAA improves insulin sensitivity as calculated by the homeostasis model assessment of insulin resistance (HOMA-IR) (*p* < 0.05), compared with the HFD + vehicle treated group (Figure 1C).

### 3.2. The Plasma and Hepatic Lipids Content of Mice Exposed to a High-Fat-Diet Feeding and Indole-3-Acetic Acid Treatment

**Figure 2:**
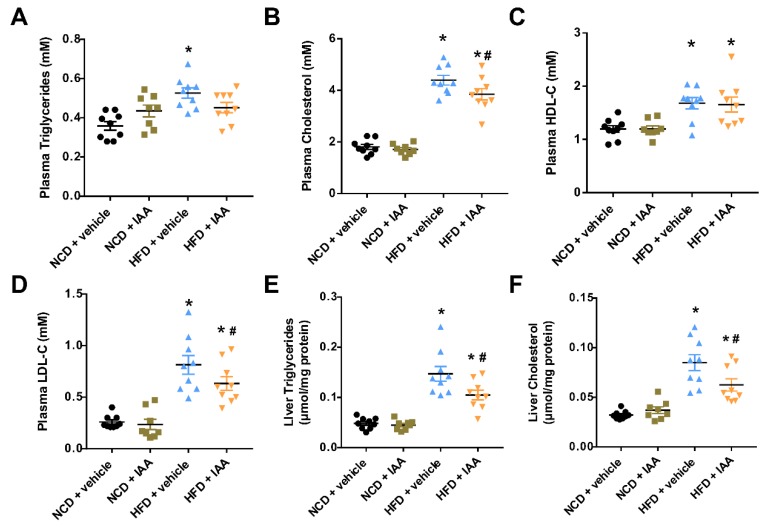
Lipid contents in the plasma and liver of vehicle or indole-3-acetic acid (IAA)-treated mice subjected to normal chow diet (NCD) or high-fat diet (HFD). (**A**–**D**) display plasma total triglycerides, total cholesterol, HDL-C, and LDL-C, respectively. (**E**,**F**) show the levels of liver triglyceride and cholesterol. Results are described as the mean ± standard error of the mean. *n* = 8–9. * *p* < 0.05 vs. NCD + vehicle; ^#^ *p* < 0.05 vs. HFD + vehicle.

To assess the effect of IAA on HFD-induced alteration in lipid metabolism, we determined the lipid profiles in the plasma and liver. As shown in Figure 2A–D, plasma total triglycerides, cholesterol, HDL-C, and LDL-C were significantly increased in mice fed with HFD compared with mice subjected to NCD feeding (*p* < 0.05). By contrast, administration with IAA exhibited a reduction in the plasma content of total cholesterol and LDL-C, compared with those in the vehicle-treated HFD group (*p* < 0.05). Furthermore, compared with mice fed a NCD, hepatic total triglycerides and cholesterol levels were increased in mice fed a HFD (*p* < 0.05). Remarkably, treatment of IAA significantly reduced the total triglycerides and cholesterol levels in the liver of mice fed with HFD (*p* < 0.05) (Figure 2E,F).

### 3.3. Indole-3-Acetic Acid Alleviates Liver Injury in Mice Fed with a High Fat Diet

**Figure 3:**
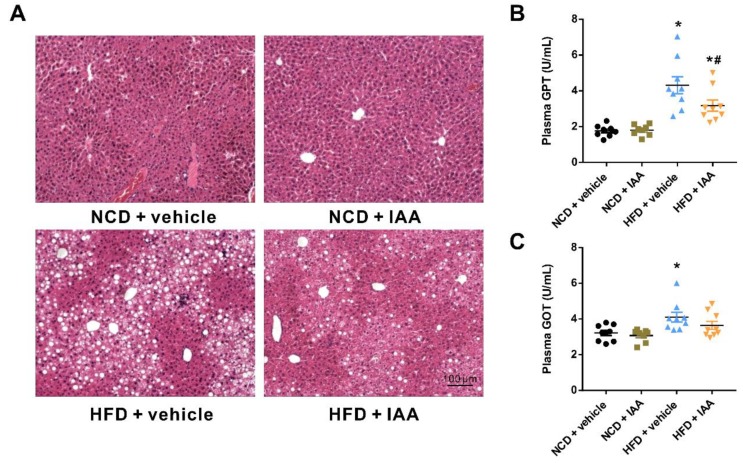
Protective effects of indole-3-acetic acid (IAA) on high-fat diet (HFD)-induced liver injury. (**A**) Representative images of hematoxylin & eosin staining results for liver tissue sections. Scale bar represents 100 µm. (**B**) Plasma glutamic-pyruvic transaminase (GPT) and (**C**) glutamic oxalacetic transaminase (GOT) levels. Data are presented as the mean ± standard error of the mean. *n* = 8–9. * *p* < 0.05 vs. NCD + vehicle; ^#^ *p* < 0.05 vs. HFD + vehicle.

Results from H&E staining of the histopathological section indicated that HFD led to marked macrovesicular steatosis combined with hepatocellular ballooning in liver tissue, which was improved by IAA treatment as shown in Figure 3A. In addition, IAA attenuated lobular inflammation induced by HFD feeding, as presented by the alleviation of the features including the infiltration of F4/80 positive Kupffer cells/macrophages (see Section 3.6) and the aggregation of small foci of mononuclear cells (Figure 3A) in liver tissues. Liver damage was also evaluated by the content of plasma glutamic-pyruvic transaminase (GPT) and glutamic oxalacetic transaminase (GOT). Mice with HFD feeding exhibited a markedly elevated level of plasma GPT and GOT, compared with the NCD group (*p* < 0.05) (Figure 3B,C). By contrast, IAA administration significantly suppressed the increase in plasma GPT of mice receiving HFD (*p* < 0.05).

### 3.4. The Regulation of Indole-3-Acetic Acid on the Expression of Genes Linked with Lipid Metabolism in Liver

**Figure 4:**
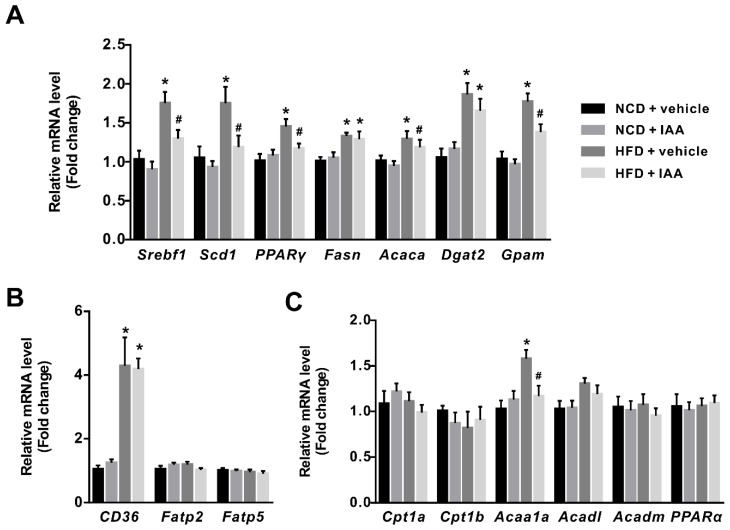
Effect of indole-3-acetic acid (IAA) on genes involved in lipid metabolism in liver of mice fed with normal chow diet (NCD) or high-fat diet (HFD). The relative expression levels of genes related to (**A**) lipogenesis, (**B**) fatty acid uptake, and (**C**) β-oxidation of fatty acids were quantified by RT-qPCR. The bar chart presents mean ± standard error of the mean of the fold change values acquired from at least 6 biologically replicates for each gene. * *p* < 0.05 vs. NCD + vehicle; ^#^ *p* < 0.05 vs. HFD + vehicle.

To further illuminate the mechanism underlying the protective role of IAA against HFD-induced hepatic steatosis, the expression of genes associated with lipid metabolism in the liver were detected by RT-qPCR assay. Exposure of mice to HFD induced a significant elevation in the expression of sterol regulatory element binding-protein 1 (*Srebf1*), steraroyl coenzyme decarboxylase 1 (*Scd1*), peroxisome proliferator-activated receptor gamma (*PPARγ*), fatty acid synthase (*Fasn*), acetyl-CoA carboxylase 1 (*Acaca*), diacylglycerol O-acyltransferase 2 (*Dgat2*), and glycerol-3-phosphate acyltransferase, mitochondrial (*Gpam*), which were related to lipogenesis (*p* < 0.05) (Figure 4A). Notably, IAA treatment suppressed a HFD-induced increase in mRNA abundance of *Srebf1*, *Scd1*, *PPARγ*, *Acaca*, and *Gpam* (*p* < 0.05). Additionally, the up-regulation of cluster of differentiation 36 (*CD36*) not fatty acid transport protein 2 (*Fatp2*) and fatty acid transport protein 5 (*Fatp5*), which function to regulate fatty acid uptake in hepatic tissues were observed in HFD feeding mice when compared with the NCD-fed mice (*p* < 0.05) (Figure 4B). The mRNA levels of genes involved in β-oxidation of fatty acid such as carnitine palmitoyltransferase-1a (*Cpt1a*), carnitine palmitoyltransferase-1b (*Cpt1b*), acyl-CoA dehydrogenase long chain (*Acadl*), acyl-CoA dehydrogenase medium chain (*Acadm*), and peroxisome proliferator-activated receptor α (*PPARα*) in HFD mice showed no significant difference compared with the control group, except for acetyl-Coenzyme A acyltransferase 1A (*Acaa1a*) (*p* < 0.05) (Figure 4C). In comparison, the elevation of *Acaa1a* in response to HFD was repressed by IAA treatment (*p* < 0.05) (Figure 4C).

### 3.5. Indole-3-Acetic Acid Attenuates High Fat Diet-Induced Oxidative Stress in Hepatic Tissue

**Figure 5:**
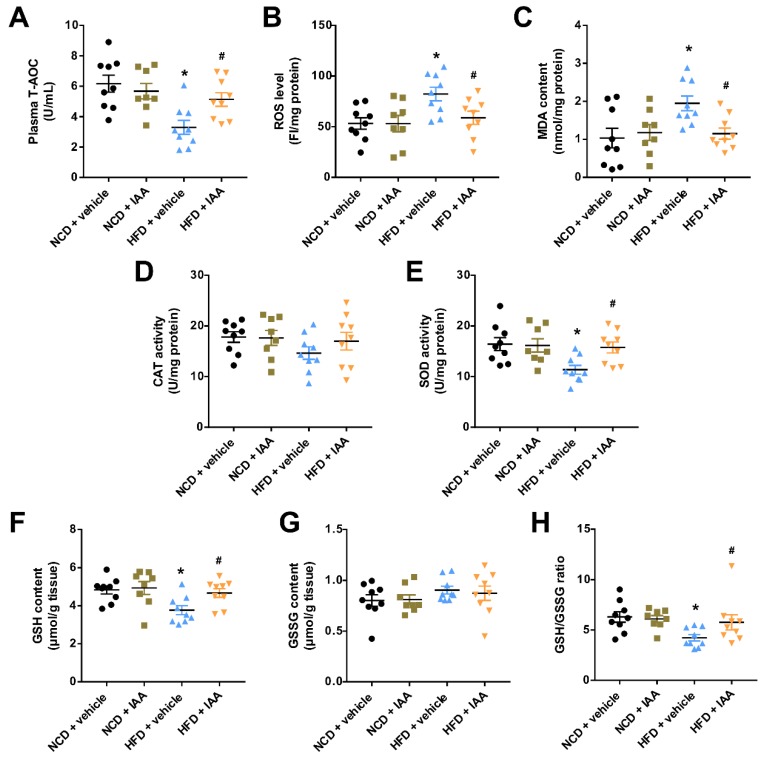
Changes in oxidative stress markers in mice exposed to high-fat diet (HFD) and indole-3-acetic acid (IAA) treatment. (**A**) plasma total antioxidant capacity (T-AOC), (**B**) and (**C**) liver reactive oxygen species (ROS) and malondialdehyde (MDA) level, F, fluorescence intensity. (**D**,**E**) liver superoxide dismutase (SOD) and catalase (CAT) activity. (**F**,**G**) the content of glutathione (GSH) and oxidized glutathione (GSSG), and (**H**) the ratio of GSH/GSSG. Values are shown as the mean ± standard error of the mean. * *p* < 0.05 vs. NCD + vehicle; ^#^ *p* < 0.05 vs. HFD + vehicle.

To evaluate the effects of IAA on oxidative stress in the hepatic tissue of mice exposed to HFD, we determined the levels of reactive oxygen species (ROS) and lipid peroxidation product as well as the activity or content of enzymes that are known to be key regulators in maintaining redox homeostasis. The mice subjected to HFD displayed significantly increased ROS and malonaldehyde (MDA) content in liver tissue compared with those exposed to NCD (*p* < 0.05). These alterations were suppressed by administration with IAA (*p* < 0.05) (Figure 5B,C). In contrast, compared with the mice fed NCD, plasma total antioxidant capacity (T-AOC) and the hepatic activity of superoxide dismutase (SOD) were reduced in mice following HFD feeding, which was restored by IAA treatment (*p* < 0.05) (Figure 5A,E). Consistently, feeding with HFD significantly decreased the hepatic glutathione (GSH) level and the ratio of GSH to oxidized glutathione (GSSG), while mice given IAA noticeably prevented these effects (*p* < 0.05) (Figure 5F,H). No significant difference was observed between the groups in terms of catalase (CAT) activity and GSSG content (Figure 5D,G). Taken together, the data above support the idea that IAA possesses a role in the alleviation of HFD-induced oxidative stress in hepatic tissue.

### 3.6. Indole-3-Acetic Acid Mitigates Hepatic Inflammatory Stress Induced by a High-Fat Diet

**Figure 6:**
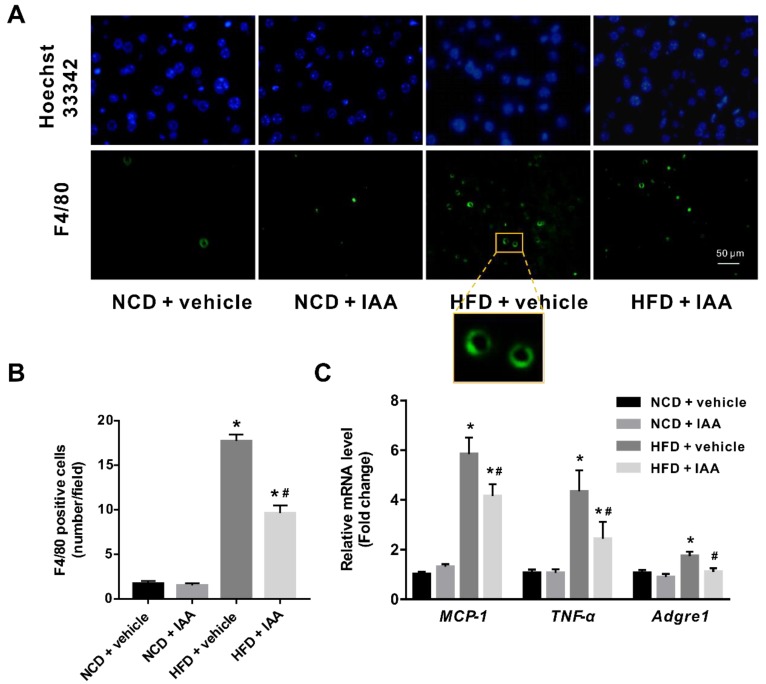
Inflammatory stress in liver of mice induced by high-fat diet (HFD) was ameliorated by indole-3-acetic acid (IAA) treatment. (**A**) Immunofluorescence analysis for F4/80 antigen in liver tissue section. Scale bar represents 50 µm. (**B**) Count of F4/80 positive cells per field (at least six field per mice) (**C**) Relative mRNA abundance of *MCP-1*, *TNF-α*, and *Adgre1*. Values are presented as the mean ± standard error of the mean. *n* = 6. * *p* < 0.05 vs. NCD + vehicle; ^#^ *p* < 0.05 vs. HFD + vehicle.

As we observed inflammatory features in the histological examination of our NAFLD model, we then analyzed hepatic inflammatory mediators in NCD or HFD feeding mice exposed to vehicle or IAA treatment. As shown in Figure 6A,B, HFD mice displayed significantly increased infiltration of activated macrophages as evidenced by F4/80 positive cells compared with that of NCD mice (*p* < 0.05). In contrast, treatment with IAA reduced the number of activated macrophages that infiltrated into hepatic tissue induced by HFD feeding (*p* < 0.05). In line with the results from immunofluorescence, RT-qPCR showed that mice subjected to HFD exhibited an upregulated adhesion G protein-coupled receptor E1 (*Adgre1*) mRNA abundance, which was suppressed by IAA administration (*p* < 0.05). Similarly, IAA treatment in mice fed HFD also gave rise to a marked reduction in the mRNA level of inflammatory cytokines including monocyte chemoattractant protein-1 (*MCP-1*) and tumor necrosis factor-α (*TNF-α*), compared with that of vehicle-treated HFD mice (*p* < 0.05) (Figure 6C). Collectively, these data suggest that IAA relieves HFD-induced inflammatory stress in hepatic tissue.

## 4. Discussion

Growing evidence has revealed the modulation of gut microbiota-derived metabolites in the development and progression of NAFLD. In the present study, we clarified that IAA, a gut bacteria metabolite derived from tryptophan, conduces to mitigate the severity of hepatotoxicity in mice exposed to HFD feeding, as evidenced by the amelioration in insulin resistance, lipid metabolism, oxidative stress, and inflammation, corroborating IAA is a potential molecule that is beneficial to relieving NAFLD. Our results provide the first in vivo evidence that supports the protective role of IAA in NAFLD.

In our study, mice fed with HFD presented with weight gain, insulin resistance, hyperglycemia, and dyslipidemia, while these effects except for weight gain were alleviated by treatment with IAA which was confirmed as an AhR ligand [17]. Consistently, the AhR ligand FICZ has been reported to improve insulin resistance, glucose metabolism, and the serum total cholesterol level without affecting body weight gain in HFD-fed mice [23]. An elevated concentration of GPT (also known as ALT) released into the blood is well known as a crucial indicator for liver damage [24]. The increased level of GPT in response to HFD feeding was attenuated following IAA treatment, suggesting the protective role of IAA to liver injury. HFD-induced hepatic steatosis in mice has been observed in plenty of studies [25,26,27]. Administration with IAA in mice exposed to HFD relieved hepatic steatosis as shown in our work, indicating the protective role of IAA against NAFLD in an experimental model of mice. In line with our study, alcohol feeding-induced liver steatosis in mice was obviously mitigated by the daily administration of IAA [28]. In addition, results from the hepatocyte cell line of mice reported that lipid accumulation in AML12 cells subjected to free fatty acids or pro-inflammatory cytokine was reversed by IAA incubation [20]. This may be associated with the negative regulation of gene expression related to de novo lipogenesis including Srebf1, Acaca, and Fasn in response to IAA or AHR agonist β-naphthoflavone, as observed in previous in vitro or in vivo studies, respectively [20,29]. Similar results in our current in vivo study first showed that following HFD exposure, the upregulation of the lipogenesis genes involving Srebf1, Scd1, PPARγ, Acaca, and Gpam was abated by IAA treatment, thereby bringing about a reduction in hepatic triglyceride accumulation.

Oxidative stress has increasingly emerged as the pivotal factor in the development and progression of NAFLD [10,30]. ROS accelerates NAFLD through the induction of lipid peroxidation and the promotion of insulin resistance, lipid accumulation, and inflammation [31]. Results observed in our study evidenced that the hepatic levels of ROS and lipid peroxidation product MDA in the HFD group were significant increased, which was concurrent with increased macrophages (F4/80-positive cells) that may expedite ROS generation and inflammatory responses. Intriguingly, IAA was capable of reducing ROS and the MDA level as well as the infiltration of activated macrophages in the liver of mice fed with HFD. An earlier study has pointed out that IAA appears to be an effective scavenger of free radical compounds [18]. Likewise, Kim et al. reported that hydrogen peroxide-induced oxidative damage in human dental pulp stem cells (hDPSCs) was notably rescued by IAA treatment [19]. Other indications of oxidative stress that emerges in the liver of mice with HFD feeding were shown by the depressed antioxidative defense systems [32,33,34]. In this regard, we observed a reduction in SOD activity and the GSH level in HFD-fed mice, which were reversed by IAA administration. Consistently, the protective effects of IAA against hepatic oxidative stress via the elevation in the expression of antioxidative enzymes has been demonstrated in diethylnitrosamine-treated mice [35]. All the aforementioned evidence supports the potential antioxidative activity of IAA, which contributes to the scavenging of excessive ROS produced in the liver, thus attenuating the severity of NAFLD.

Inflammatory responses in hepatic tissue is regarded as the primary cause of impairment to hepatic tissue, which leads to serious fibrogenesis and eventually hepatocellular carcinoma with the progression of NAFLD [36]. Our present study showed an increase in the infiltration of activated macrophages and the expression of TNF-α and MCP-1 in hepatic tissue of mice subjected to HFD feeding. Liver Kupffer cells and their recruited macrophages in response to inflammatory responses differentiate into activated macrophages that express the F4/80 antigen, which promotes the expression of inflammatory cytokines and the generation of ROS [37,38]. MCP-1 is a key chemokine that functions primarily to recruit monocytes and macrophages into the sites of inflammation [39]. Following HFD feeding, mice may display hepatic inflammation accompanied by elevated MCP-1 expression as evidenced by considerable previous reports [25,40,41,42]. Additionally, the upregulation of MCP-1 expression in the liver has also been observed in HFD-fed mice exhibiting hepatic steatosis without marked inflammatory lesions [43], suggesting MCP-1 plays crucial roles in the pathogenesis of NAFLD. A recent study by Kakino et al. indicated the pivotal role of TNF-α in the development and progression of NAFLD, which is driven by the upregulation of critical molecules linked to hepatic lipid metabolism, inflammatory cytokines and fibrosis [44]. As a pro-inflammatory cytokine, TNF-α triggers a cytotoxic immune response, thereby leading to liver tissue injury. Consistent with our study, the increased abundance of TNF-α expression in the hepatic tissue of mice exposed to HFD has been verified in numerous research studies [40,41,42]. Interestingly, HFD-induced elevation in the expressions of F4/80, MCP-1, and TNF-α in the liver of mice were alleviated following IAA treatment. An in vitro study by Krishnan et al. substantiated the efficient anti-inflammation activity of IAA on macrophages as evidenced by a reduction in TNF-α, IL-1β, and MCP-1 of the macrophage cell line exposed to palmitate and LPS [20]. Hence, IAA is beneficial to the amelioration of hepatic inflammation that presents in NAFLD mice, which may be relevant to the lightened inflammatory responses mediated by macrophages.

In summary, the findings provided by our study has verified the ameliorating effects of IAA on hepatotoxicity induced by HFD feeding in mice, which turn out to be explained by the improvement in insulin resistance, lipid metabolism, oxidative and inflammatory stress. Therefore, it is anticipated that the ingestion of foods rich in tryptophan favoring the production of IAA from gut microbiota may serve to resist the pathological processes of NAFLD.

## Supplementary Materials

The following are available online at https://www.mdpi.com/2072-6643/11/9/2062/s1, Figure S1: Effect of indole-3-acetic acid (IAA) administration on body weight, feed intake, energy intake, and liver weight of mice subjected to normal chow diet (NCD) or high-fat diet (HFD) feeding. (A) Feed intake. (B) Energy intake. (C) Body weight. (D) Liver weight. Results are presented as the mean ± standard error of the mean. *n* = 8–9. * *p* < 0.05 vs. NCD + vehicle, Table S1: Primer sequences used for quantitative real-time PCR (RT-qPCR).

## Abbreviations

Acaca, acetyl-CoA carboxylase 1; Acadl, acyl-CoA dehydrogenase long chain; Acadm, acyl-CoA dehydrogenase medium chain; CAT, catalase; CD36, cluster of differentiation 36; Cpt1a, carnitine palmitoyltransferase-1a; Cpt1b, carnitine palmitoyltransferase-1b; Dgat2, diacylglycerol O-acyltransferase 2; Fasn, fatty acid synthase; Fatp2, fatty acid transport protein 2; Fatp5, fatty acid transport protein 5; Gpam, glycerol-3-phosphate acyltransferase, mitochondrial; GPT, glutamic-pyruvic transaminase; GOT, glutamic oxalacetic transaminase; GSH, glutathione; GSSG, oxidized glutathione; HFD, high-fat diet; HOMA-IR, homeostasis model assessment of insulin resistance; IAA, indole-3-acetic acid; MCP-1, monocyte chemoattractant protein-1; MDA, malonaldehyde; NAFLD, nonalcoholic fatty liver disease; NCD, normal chow diet; PPARα, peroxisome proliferator-activated receptor α; PPARγ, peroxisome proliferator-activated receptor gamma; ROS, reactive oxygen species; Scd1, steraroyl coenzyme decarboxylase 1; SOD, superoxide dismutase; Srebf1, sterol regulatory element binding-protein 1; T-AOC, total antioxidant capacity; TNF-α, tumor necrosis factor-α.
