# Supplementary material for: Indole-3-Acetic Acid Alleviates Nonalcoholic Fatty Liver Disease in Mice via Attenuation of Hepatic Lipogenesis, and Oxidative and Inflammatory Stress
Source: Nutrients. 2019 Sep 3;11(9):2062. doi: 10.3390/nu11092062 (PMC6769627; doi:10.3390/nu11092062)
Supplement: Supplementary file 1 [file nutrients-11-02062-s001.pdf]

Supplementary Table S1. Primer sequences used for quantitative real-time PCR (RT-qPCR)

Gene	Accession No.	Primers (5′-3′)	Product size (bp)
Acaa1a	NM_130864.3	F: CCTGACTCCTATGGGGATGA<br>R: CCCTTGTCATCCAGGACAGT	174
Acaca	NM_133360.2	F: GAGAGGGGTCAAGTCCTTCC<br>R: ACATCCACTTCCACACACGA	152
Acadl	NM_007381.4	F: TCACCACACAGAATGGGAGA<br>R: ACGCTTGCTCTTCCCAAGTA	155
Acadm	NM_007382.5	F: AGGTTTCAAGATCGCAATGG<br>R: GCGAGCAGAAATGAAACTCC	168
Adgre1	NM_001355722.1	F: ATACCCTCCAGCACATCCAG<br>R: AGTTTGCCATCCGGTTACAG	159
CD36	NM_001159558.1	F: GCTGTGTTTGGAGGCATTCT<br>R: TGGGTTTTGCACATCAAAGA	162
Cpt1a	NM_013495.2	F: CCAGGCTACAGTGGGACATT<br>R: AAGGAATGCAGGTCCACATC	100
Cpt1b	NM_009948.2	F: CCCATGTGCTCCTACCAGAT<br>R: CCTTGAAGAAGCGACCTTTG	130
Dgat2	NM_026384.3	F: CTTCCTGGTGCTAGGAGTGG<br>R: GCCAGCCAGGTGAAGTAGAG	96
Fasn	NM_007988.3	F: CCCTTGATGAAGAGGGATCA<br>R: CAAGGCGTTAGGGTTGACAT	99
Fatp2	NM_011978.2	F: ATCAACTGGGCCTACGACAG<br>R: AGCAGAGACTTGGCACGAAT	142
Fatp5	NM_009512.2	F: TCGGATCTGGGAATTCTACG<br>R: AAGCTCAAAGGGAGTCAGCA	121
GAPDH	NM_001289726.1	F: AACTTTGGCATTGTGGAAGG<br>R: GGATGCAGGGATGATGTTCT	132
Gpam	NM_008149.4	F: TTATCACCAGGACGGAAAGG<br>R: TTTTCACAGCGTTCTTCACG	78
MCP-1	NM_011333.3	F: TCCCAATGAGTAGGCTGGAG<br>R: TCTGGACCCATTCCTTCTTG	126
PPARα	NM_001113418.1	F: TGCAAACTTGGACTTGAACG<br>R: GATCAGCATCCCGTCTTTGT	106
PPARγ	NM_001127330.2	F: GATGGAAGACCACTCGCATT<br>R: CAACCATTGGGTCAGCTCTT	116
Scd1	NM_009127.4	F: GCGATACACTCTGGTGCTCA<br>R: CCCAGGGAAACCAGGATATT	117
Srebf1	XM_011248845.2	F: TACTTCTTGTGGCCCGTACC<br>R: TCAGGTCATGTTGGAAACCA	129
TNF-α	NM_001278601.1	F: GCTGAGCTCAAACCCTGGTA<br>R: AGTACTTGGGCAGATTGACCT	100

Acaca, acetyl-CoA carboxylase 1; *Acadl*, acyl-CoA dehydrogenase long chain; *Acadm*, acyl-CoA dehydrogenase medium chain; *CD36*, cluster of differentiation 36; *Cpt1a*, carnitine palmitoyltransferase-1a; *Cpt1b*, carnitine palmitoyltransferase-1b; *Dgat2*, diacylglycerol O-acyltransferase 2; *Fasn*, fatty acid synthase; *Fatp2*, fatty acid transport protein 2; *Fatp5*, fatty acid transport protein 5; *GAPDH*, glyceraldehyde-3-phosphate dehydrogenase; *Gpam*, glycerol-3-phosphate acyltransferase, mitochondrial; *MCP-1*, monocyte chemoattractant protein-1; *PPARα*, peroxisome proliferator-activated receptor α; *PPARγ*, peroxisome proliferator-activated receptor gamma; *Scd1*, steraroyl coenzyme decarboxylase 1; *Srebf1*, sterol regulatory element binding-protein 1; *TNF-α*, Tumor necrosis factor-α.

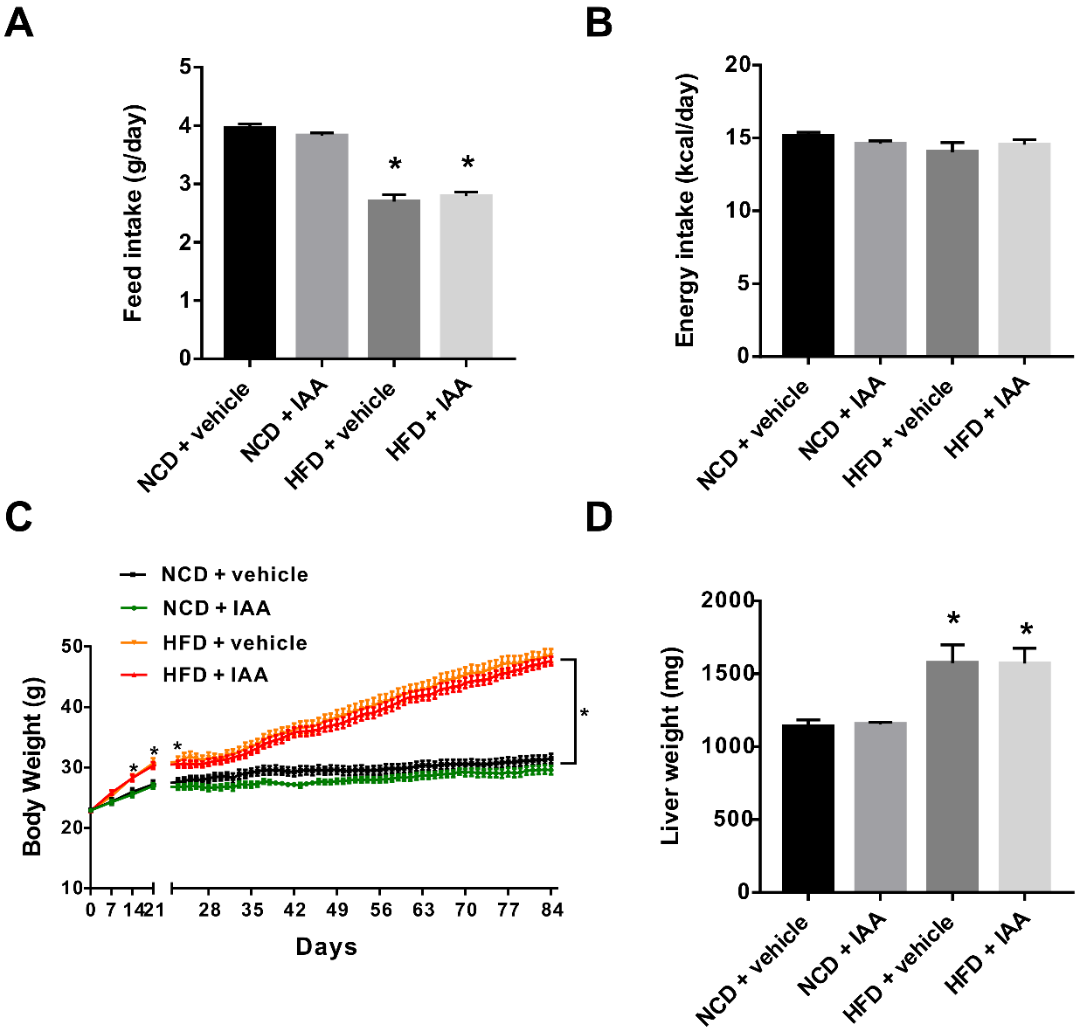

Supplementary Figure S1. Effect of indole-3-acetic acid (IAA) administration on body weight, feed intake, energy intake, and liver weight of mice subjected to normal chow diet (NCD) or high-fat diet (HFD) feeding. (A) Feed intake. **(B)** Energy intake. **(C)** Body weight. **(D)** Liver weight. Results are presented as the mean ± standard error of the mean. n = 8-9. *$p < 0.05$ vs. NCD + vehicle.
